# 5G EMF Exposure at 3.6 GHz in Greece Using Data From Frequency‐Selective Monitoring Sensors

**DOI:** 10.1002/bem.70008

**Published:** 2025-05-08

**Authors:** Serafeim Iakovidis, Athanasios Manassas, Christos Apostolidis, Theodoros Samaras

**Affiliations:** ^1^ CIRI—Center for Interdisciplinary Research and Innovation Aristotle University of Thessaloniki Thermi Greece

## Abstract

The introduction of 5G networks has raised public concerns about potential changes in environmental electromagnetic field (EMF) exposure. This study analyzes continuous monitoring data collected over 2 years (August 2022–October 2024) from 13 frequency‐selective monitoring sensors located in Greece's five largest cities. Focusing on the 3.6 GHz band, we evaluated trends and weekly variations in EMF levels. Results indicated a gradual increase in EMF exposure at 3.6 GHz, driven by the growing penetration of 5G infrastructure and devices. Notably, this band exhibited higher maximum‐to‐median power density ratios compared to other frequency bands, attributable to active antenna systems' characteristics and traffic variations. Applying the ICNIRP 2020 guidelines, we found that 30‐min averaged values significantly reduced these variations. All measured EMF levels, including maximum values, remained well below Greek and international safety limits. These findings, especially the increasing trend identified for the EMF levels, underscore the importance of continuous monitoring networks for assessing EMF exposure to existing and emerging telecommunications networks and ensuring compliance with safety standards.

## Summary

- Over the last 2 years, 5G EMF levels at the 3.6 GHz frequency band increased due to network expansion but stayed well below ICNIRP and Greek reference levels.
- The 3.6 GHz band showed much higher maximum‐to‐median E‐field variability than other frequency bands, driven by active antenna systems and traffic variations.
- Using 30‐min averaging, as recommended by ICNIRP (2020), significantly reduced exposure variability at 3.6 GHz, aligning it with the one observed in frequency bands used by cellular networks of previous generations.

## 1 Introduction

The introduction of fifth generation (5G) networks in late 2018 has offered enhanced mobile connectivity in terms of speed (high‐data rates), synchronization (reduced latency) and number of connected devices (IoT). The frequency bands assigned to 5G networks are largely divided into frequency range 1 (FR1, 410‐7125 MHz) and frequency range 2 (FR2, 24.25–71 GHz) (TSGR 2024). While the FR2 frequency band has not yet been adopted by most mobile network operators worldwide, and especially in Europe (5G Observatory Biannual Report 2023), the use of FR1 is continuously rising since 2018 in terms of auctioned spectrum (5G Observatory Biannual Report 2023), number of installed base stations (BS) (TSGR 2024) and data traffic (Ericsson 2024). Within the FR1, the recently introduced 700 MHz and 3.6 GHz bands are used for 5G network deployment, while a significant number of 5G BS operates at the intermediate frequency bands, along with 4G/2G, applying dynamic spectrum sharing (DSS). From an electromagnetic field (EMF) exposure perspective, the 3.6 GHz band has gained attention, not only for the aforementioned reasons but also due to the deployment of active antenna systems. The challenges in exposure assessment imposed by features of such antenna systems (e.g., beamforming/beam steering) have led to a significant number of published works (Aerts 2021; Migliore et al. 2021; Korkmaz 2024; Shi et al. 2024; Bilson et al. 2024) and standards (e.g., IEC 2022) trying to address them. While the variability of EMF levels pertaining to legacy networks (i.e., 2G to 4G) is dominantly attributed to network traffic variations over time, EMF level fluctuations of 5G (whenever active antennas are deployed) are induced both by time and space variation of the network's users/usage. Therefore, the investigation of the variations of EMF levels from 5G networks and the identification of differences compared to other legacy cellular and/or broadcasting systems is important from an EMF exposure assessment point of view. While such studies have already been performed for 2G to 4G and broadcasting networks (Manassas et al. 2012, 2023; Joseph et al. 2009; Joseph and Verloock 2010; Aerts et al. 2018; Joshi et al. 2015), there is no, to the best of the authors' knowledge, published work dealing with 5G networks using active antennas. In this study, for the first time, continuous environmental EMF measurement data of 5G networks at the frequency band of 3.6 GHz and for an extensive time period of 2 years (August 2022–October 2024) are presented and analyzed. Trends in exposure are identified and variations over shorter periods are evaluated and compared to older generations of cellular networks (i.e., 2G to 4G).

## 2 Materials and Methods

### 2.1 Data Collection

**Figure 1:**
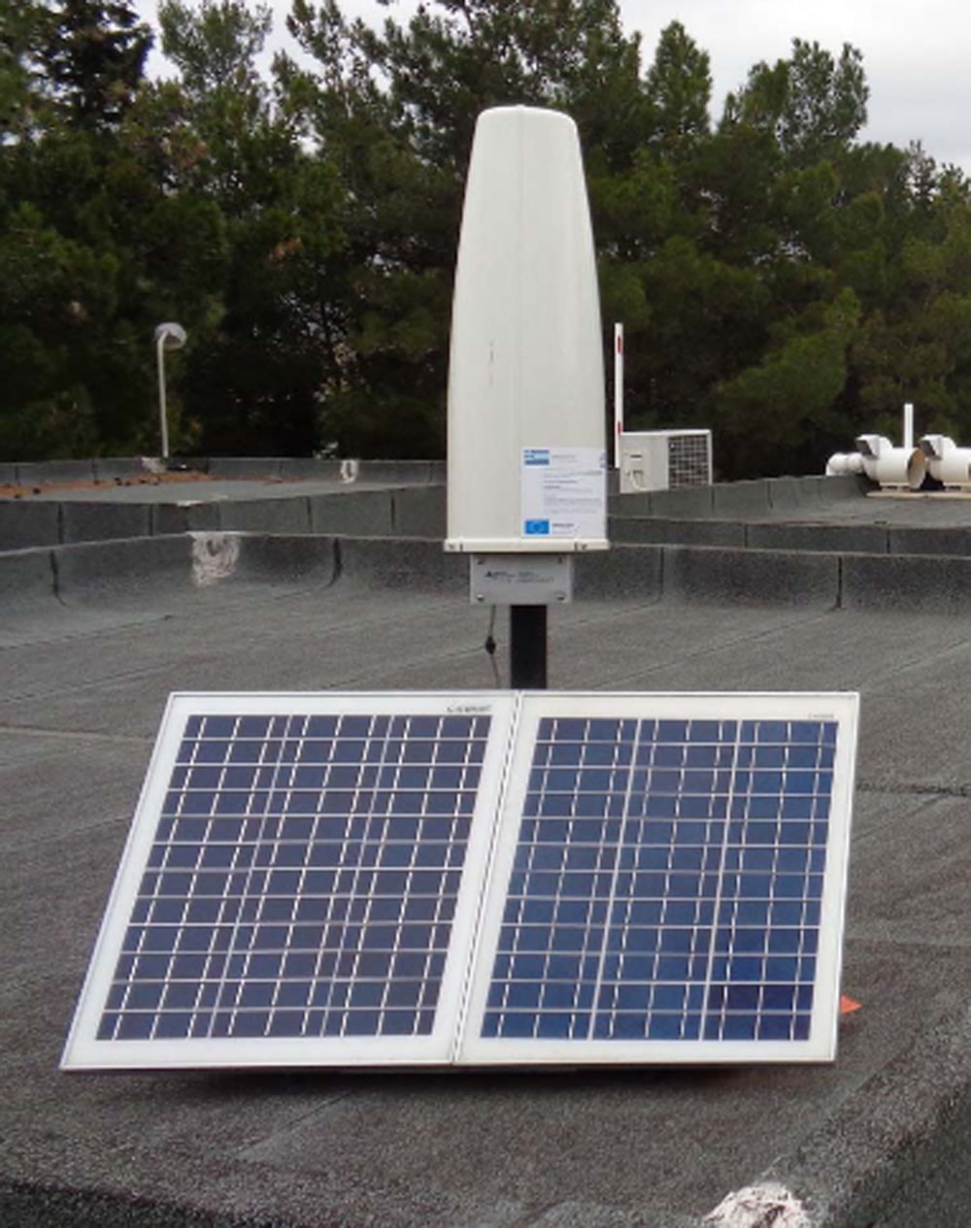
Installation of a frequency‐selective monitoring sensor (AMS‐8061/G, Narda) on a rooftop of a public building (Karastergios et al. 2020).

The data set includes continuous monitoring EMF data from 13 different frequency‐selective monitoring sensors located in the five largest Greek cities, that is, Athens (8), Thessaloniki (2), Patra (1), Heraklion (1), and Larisa (1). The data set used is provided by the National Observatory of Electromagnetic Fields (NOEF) (Christopoulou et al. 2024; Karastergios et al. 2020) and is publicly available from the NOEF's portal (National Observatory of Electromagnetic Fields n.d.). It consists of continuous 6‐min average (root mean square, RMS) measurements of the electric field (E‐field) in the 13 different locations mentioned above. A detailed description of the measurements (e.g., hardware used, frequency range, band description, uncertainty, etc.) can be found in Karastergios et al. (2020). Frequency selective monitoring sensors (AMS‐8061/G, Narda STS, Pfullingen, Germany), Figure 1) were used to acquire the measurement data. The monitoring sensors are capable of measuring RMS E‐field (V/m) at 20 different frequency bands (Table 1) included in the frequency range of 100 kHz–6 GHz. The expanded measurement uncertainty is less than 4 dB (Karastergios et al. 2020) and the sensitivity is 0.01 V/m (AMS‐8061 Datasheet).

**Table 1:** Frequency bands used in the frequency‐selective EMF monitoring sensors (Karastergios et al. 2020).

Band no	Frequency range (MHz)	Band no	Frequency range (MHz)
1	0.1–2	11	1436–1710
2	2–87.5	12	1710–1920
3	87.5–108	13	1920–2170
4	108–174	14	2170–2500
5	174–369	15	2500–2690
6	369–470	16	2690–3400
7	470–790	17	3400–3770
8	790–876	18	3770–5470
9	876–960	19	5470–5725
10	960–1436	20	5725–6000

### 2.2 Data Analysis

Out of the 20 different frequency bands of Table 1, eight (8) were considered for further analysis: Band no 3 (FM broadcast), band no 8 (DVB‐T broadcast and mobile services) and band nos. 9, 12, 13, 15, 17 (mobile services). The considered frequency bands include the majority of EMF emissions in an outdoor urban environment. The rest were excluded for brevity, both in analyzing and presenting the results.

The measurements were obtained from August 29, 2022 to October 16, 2024 (i.e., in a period of 112 weeks). Some monitoring sensors have missing data due to their temporary removal from their installation site for service or recalibration purposes, so linear interpolation was applied for data imputation. Processing and imaging of the data was performed using code developed in MATLAB (The MathWorks Inc., Natick, MA, USA).

The data were aggregated over every week, thus generating 112 weeks in the above measurement period (with the 112th week containing 2 days only). Each monitoring sensor logs 1680 (every 6 min) values of the RMS E‐field per full 7‐day week. The weekly median, 95th‐percentile, 99th‐percentile, and maximum of these 6‐min averages were evaluated. To evaluate the temporal variability of the E‐field levels, different metrics were found in the literature: (a) $\frac{E_{\max}-E_{\min}}{E_{\min}}\%$, in Manassas et al. (2012) and (b) $20∙\log_{10}\left(\frac{E_{\max}}{E_{\min}}\right)[\mathrm{dB}]\;\text{or}\;20∙\log_{10}\left(\frac{E_{P95}}{E_{P5}}\right)[\mathrm{dB}]$ in Joseph and Verloock (2010) and Aerts et al. (2018) and (c) $R=\frac{E_{\mathrm{median}}}{E_{\max}}$ in Joseph et al. (2009). We followed the approach presented in Joseph et al. (2009) with a minor change, by selecting the ratio of weekly maximum to median power density value (i.e., $\frac{1}{R^{2}}$). The inverse of R ratio was selected to emphasize the changes in weekly maxima. Then, the inverse was squared, because power density in free space $S\approx \frac{E^{2}}{377}$ is proportional to the square of E‐field in the far field. Consequently, this metric is the closest proxy to the peak‐to‐average emitted power density towards the monitoring sensor. Although the concept of peak‐to‐average power is more important for short‐time variations, it can still capture the implementation of active antenna beamforming within a 6‐min interval.

To evaluate the trend of weekly median RMS E‐field values over time across different frequency bands, along with their corresponding statistical significance, the following procedure was applied: First, the assumption of data independence was checked by calculating the lag‐1 autocorrelation for each frequency band. As noted by Wilson (2016), autocorrelation functions are widely used in time series analysis to determine whether current values are linearly related to past values. A significant autocorrelation at a given lag indicates a violation of the independence assumption and supports the use of specialized statistical methods, such as the Modified Mann‐Kendall Test (Hamed and Rao 1998) Second, due to the presence of strong autocorrelation in the weekly median E‐field values, the Modified Mann‐Kendall Test (Hamed and Rao 1998) was applied. This test accounts for serial correlation and provides a more accurate assessment of trend significance. It was applied to each time series of weekly median RMS E‐field values, across all frequency bands and for each of the 13 sensors. Finally, the results were aggregated for each frequency band. The entire procedure was implemented in MATLAB.

## 3 Results

**Figure 2:**
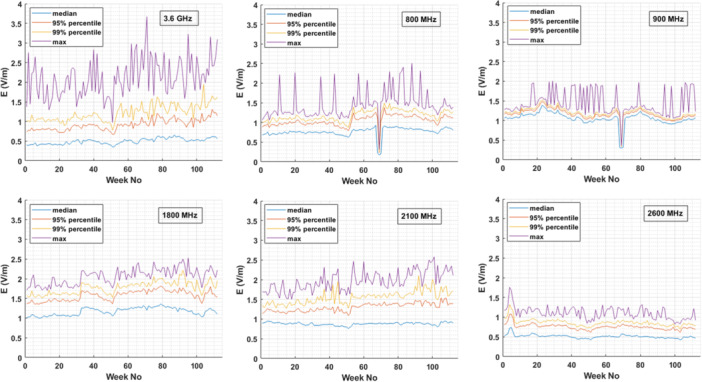
Weekly median, 95th‐percentile, 99th‐percentile and maximum 6‐min‐averaged E‐field value for six different frequency bands (800 MHz, 900 MHz, 1800 MHz, 2100 MHz, 2600 MHz, and 3.6 GHz) as logged by the frequency selective monitoring sensor located in Chalandri, Attica over a period of 2 years (i.e., August 29, 2022–October 16, 2024).

Results for one monitoring sensor placed at the rooftop of a public building located in Chalandri, Attica are plotted in Figure 2, for different frequency bands used by cellular networks (CNs). Weekly median, maximum, 95th and 99th percentile values are plotted to provide an indication of the variability of 6‐min values within a week across the different frequency bands. The plot is similar to the ones obtained from all 13 monitoring sensors used in this study and is provided here as an example of the time evolution of the E‐field.

**Figure 3:**
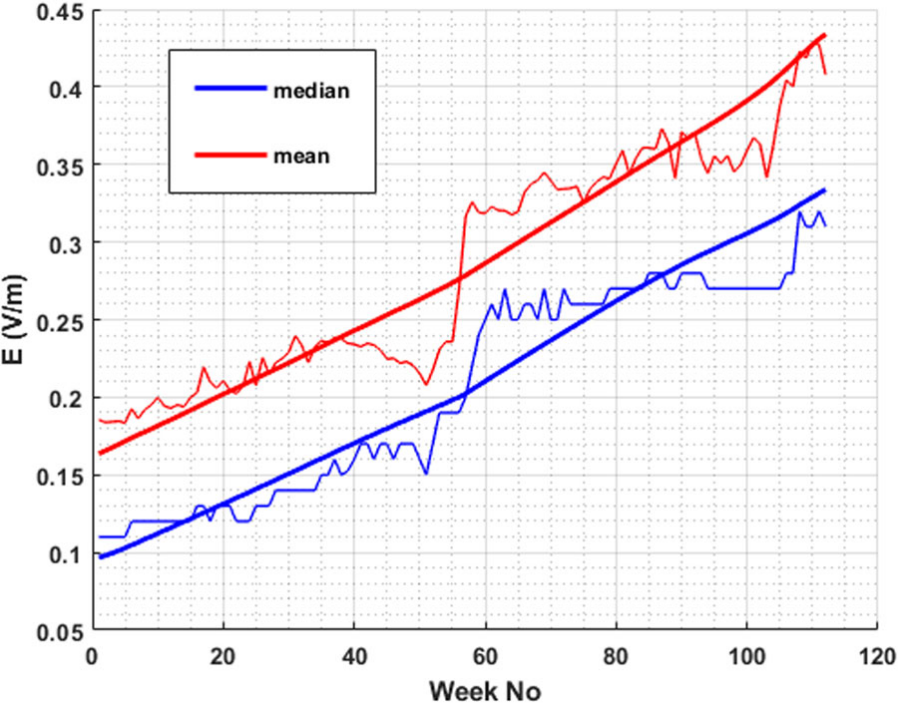
Mean (red) and median (blue) values of the distribution of the 13 monitoring sensors for the weekly median values of each sensor at the 3.6 GHz frequency band, with trendlines (thick lines).

In Figure 3, the mean and median values of the distribution of 13 different monitoring sensors'/locations' weekly median values are plotted. The corresponding trend lines (thick lines) are also included in the same figure. They are intended to provide an overall trend of the E‐field levels at 3.6 GHz frequency band, using the measurement data of this study, and were obtained in MATLAB using a function that implements singular spectrum analysis (SSA), which is a useful algorithm when the periods of the seasonal trends are unknown and the time series data are uniformly spaced.

**Figure 4:**
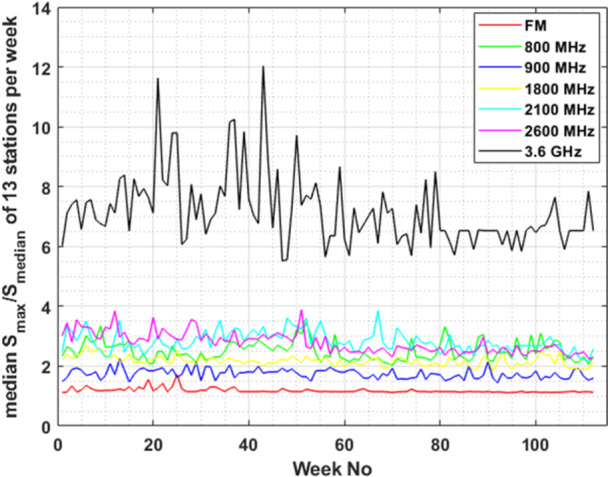
Median value of the ratio of weekly maximum to median power density value (S) for the 13 monitoring sensors. All cellular frequency bands are included for comparison. Evaluations are based on 6‐min‐averaged RMS E‐field measurements.

**Figure 5:**
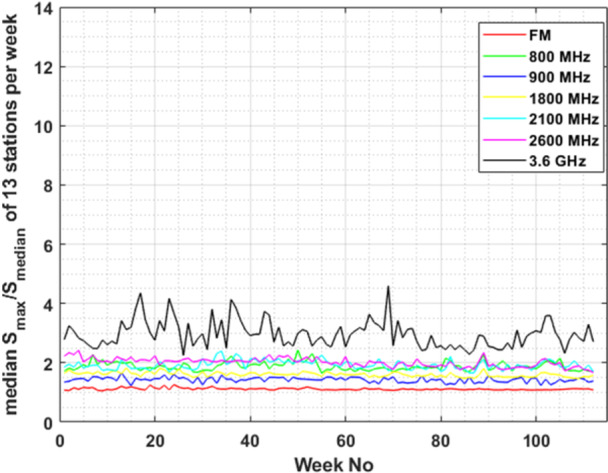
Median value of the ratio of weekly maximum to median power density value (*S*) for the 13 monitoring sensors. All cellular frequency bands are included for comparison. Evaluations are based on 30‐min‐averaged RMS E‐field measurements.

Focusing on the variability of the E‐field level within a week, across different frequency bands, we plotted (Figure 4) the median ratio of maximum to median power density values (i.e. $\frac{1}{R^{2}}$, as described in data analysis section) of the 13 different monitoring sensors/locations. The evaluation of the ratio in Figure 4 was based on the 6‐min‐averaged E‐field RMS values. Using the 30‐min‐averaged E‐field RMS values, as the latest ICNIRP guidelines suggest (ICNIRP 2020), we made the same plot (Figure 5) to highlight the impact of time‐averaging on the maximum to median ratio.

**Figure 6:**
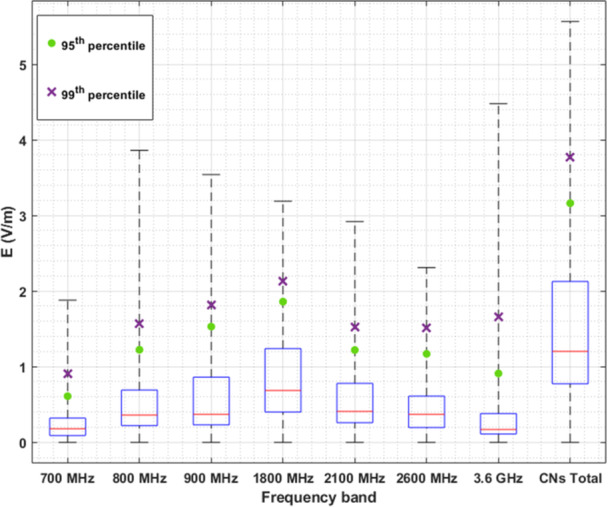
Median value (red lines), interquartile range (blue boxes), minimum and maximum values (dashed black lines), 95th percentile (green circles) and 99th percentiles (purple x's) of the distributions including all 6‐min‐averaged E‐field values for all monitoring sensors over the whole time period, plotted for each frequency band of cellular networks. The values depicted in this figure are tabulated in Table 2.

To provide a comparison of exposure across different frequency bands used by cellular networks (CNs), we plotted the median, interquartile range and min/max values of the distribution including all the 6‐min‐averaged E‐field values of all 13 different sensors over the whole time period of the study (Figure 6). The figure includes box plots to visualize the values mentioned above, as well as the 95th and 99th percentile values for every frequency band, along with a total value including emissions from all frequency bands used by cellular networks. These results are also included in Table 2 in tabular form. A comparison of the results of this study pertaining to the temporal variability with the corresponding ones of older studies (Joseph et al. 2009) in particular, is presented in Table 3. To make the comparison possible, we applied the metric used in (Joseph et al. 2009), (i.e., the R ratio), for each frequency band.

**Table 2:** Median value, interquartile range, minimum and maximum values, 95th percentile and 99th percentiles of the distributions including all 6‐min‐averaged E‐field values (V/m) for all monitoring sensors over the whole time period, for each frequency band of cellular networks.

Frequency band	median	interquartile range	95th percentile	99th percentile	max
700 MHz	0.18	[0.09, 0.32]	0.61	0.91	1.88
800 MHz	0.36	[0.22, 0.69]	1.22	1.57	3.86
900 MHz	0.37	[0.23, 0.86]	1.53	1.82	3.54
1800MHz	0.69	[0.40, 1.24]	1.86	2.13	3.19
2100 MHz	0.41	[0.26, 0.78]	1.22	1.53	2.92
2600 MHz	0.37	[0.20, 0.61]	1.17	1.51	2.31
3600 MHz	0.17	[0.11, 0.38]	0.91	1.66	4.48
CNs Total	1.20	[0.77, 2.13]	3.16	3.77	5.56

**Table 3:** E‐field levels for different frequency bands, in terms of R ratio values,^a^ as evaluated in Joseph et al. (2009) and in the current study.

Frequency band	Joseph et al. (2009)		Current study	
	Median R	[min, max] R	Median R	[min, max] R
FM	0.92	[0.82,0.92]	0.93	[0.68, 0.96]
800 MHz	‐‐‐	‐‐‐	0.63	[0.33, 0.80]
900 MHz	0.63	[0.51,0.79]	0.75	[0.59, 0.88]
1800MHz	0.70	[0.57,0.75]	0.67	[0.57, 0.86]
2100 MHz	0.60	[0.54, 0.71]	0.60	[0.45, 0.72]
2600 MHz	‐‐‐	‐‐‐	0.60	[0.41, 0.74]
3600 MHz	‐‐‐	‐‐‐	0.38	[0.18, 0.82]

^a^ Ratio of weekly E‐field median value to the week's E‐field maximum value ($R=\frac{E_{\mathrm{median}}}{E_{\max}}$).

**Figure 7:**
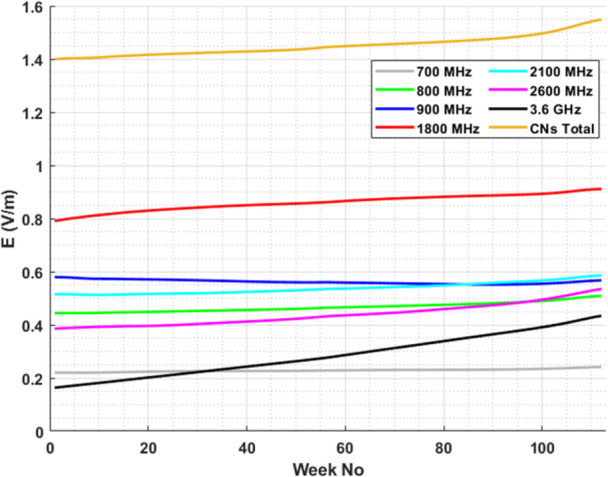
Trend of weekly mean E‐field levels across 13 measurement locations, over the whole time period of the data set, for all frequency bands used by cellular networks.

Finally, in Figure 7, the trend of weekly mean E‐field levels for different frequency bands of the cellular networks is plotted for the considered time period. To provide a measure for the statistical significance of these trends, we evaluated the individual trend of each sensor's measurements at each frequency band (Table 4). The statistical significance of each trend is also evaluated by applying the Modified Mann‐Kendal Test.

**Table 4:** Trends of 13 individual sensors across frequency bands with corresponding statistical significance.

Frequency band	Number of sensors with a statistically significant increasing trend	Number of sensors with a statistically significant decreasing trend
700 MHz	4 (7)^a^	1 (6)
800 MHz	5 (5)	6 (8)
900 MHz	1 (6)	6 (7)
1800 MHz	7 (10)	2 (3)
2100 MHz	5 (10)	1 (3)
2600 MHz	8 (10)	2 (3)
3600 MHz	12 (12)	1 (1)
CNs Total	8 (10)	2 (3)

^a^ The numbers in parentheses indicate the total number of sensors with an increasing/decreasing trend, i.e., including those for which the respective trend is not statistically significant.

## 4 Discussion

First, a gradual increase in the EMF levels of 5G at 3.6 GHz is observed during the last 2 years for individual sensors (Figure 2). This is more prominent in Figure 3, where the median and mean value for all 13 monitoring sensors' distribution of weekly median values is plotted. This is an expected result since the penetration of 5G networks in Greece is continuously rising, in terms of both installed BS and 5G‐enabled devices. According to the 5G Observatory Reports, the number of BS in Greece operating at the 3.–4‐3.8 GHz band were 247 in April 2023 (5G Observatory Biannual Report 2024) and 853 in June 2024 (5G Observatory Biannual Report 2023), while Christopoulou et al. (2024) suggested that in 2022 the number of 5G stations operating in this band was 117.

Second, seasonal variations (e.g., summer vs. winter) can also be observed. These are attributed to the movement of population from cities (where the 13 sensors are installed) to vacation locations during the summer. This movement peaks on the August 15 and adjacent days (i.e. week 51 for 2023 and week 103 for 2024) when local minima in the EMF levels are observed (Figures 2 and 3).

Third, the measured EMF levels in the 3.6 GHz band vary within a week significantly more than in any other frequency band. While the weekly median values of the E‐field at the 3.6 GHz frequency band are the lowest ones (Figure 2, top left plot, blue line), the weekly maximum values (purple line) are the highest of all frequency bands. A more representative view of all 13 monitoring sensors used in this study is provided in Figure 4. The figure shows clearly that in the 3.6 GHz frequency band the ratio of the maximum 6‐min‐averaged power density in a week can become 12 times higher than the median 6‐min‐averaged power density of that week. This maximum‐to‐median ratio (as expressed by $\frac{1}{R^{2}})$ remains lower than 4 for the other frequency bands (and close to 1, as expected, for the FM band). This difference in the maximum‐to‐median ratio can be attributed to the fact that at the 3.6 GHz frequency range active antenna systems are installed, so beamforming, beam‐steering and traffic variations can significantly change the EMF levels at a specific location over time. The higher bandwidth allocated around 3.6 GHz compared to any other band, also contributes to the larger variation observed.

Following the latest International Commission on non‐Ionizing Radiation Protection (ICNIRP) guidelines (ICNIRP 2020), we averaged the E‐field measurements to calculate the RMS values over 30 min, which is the new averaging time for the basic restriction to whole‐body specific absorption rate (SAR). This resulted in a significant reduction of the maximum‐to‐median ratio of the power density over a week for all frequency bands (Figure 5). The maximum‐to‐median ratio for the 30‐min averaged values at the 3.6 GHz frequency band, became of the same order of magnitude (≈3) as those at frequency bands of older generation cellular networks (≈2). It is important to note that all E‐field values measured by all 13 monitoring sensors over the 2‐year period, whether 6‐min or 30‐min averaged, remain significantly lower than the reference level for whole body exposure at 3.6 GHz: The highest 6‐min‐averaged power density registered over 2 years was 0.053 W/m^2^, which is much lower than both the ICNIRP and the Greek reference levels for general public exposure, that is, 10 and 6 W/m^2^, respectively. It's worth mentioning that any E‐field value used in this study corresponds to the 6‐min RMS, that is, it is immediately comparable to established safety limits in Greece. However, the significantly higher E‐field variability at the 3.6 GHz frequency band, compared to other cellular network bands, highlights the need for application of “extrapolation to the maximum” techniques when 6‐min spot (in situ) measurements are performed for compliance assessment. This point should also be considered when exposure assessment studies are performed.

Taking a closer look at both Figures 3 and 4, we observe that although the E‐field mean and median values follow an upward trend at 3.6 GHz (Figure 3) over time, the corresponding variability exhibits a rather stable or even downward trend (Figure 4). One reason for this observation could be that the number of 5G subscriptions (i.e., users) is continuously rising in the selected time period (Ericsson 2024). As already shown in Joshi et al. (2024) and Di Paola (2024), an increase in the number of connected users leads to a decrease in the maximum realized gain of the BS antenna (i.e., the emitted power is more uniformly distributed across different angles) which in turn leads to a decrease in the ratio of maximum to median measured values. Moreover, since the examined time period coincides with the initial deployment of 5G networks at 3.6 GHz, it is highly probable that the number of BS in close proximity to the measurement locations (i.e., significantly contributing to the measured E‐field levels), increases during this period. While this fact leads to an increase in median values (Figure 3), the corresponding impact on maximum values is lower, as mentioned in § B.9.6 (ICE 2022): “The probability that multiple independent transmitter systems are delivering the actual maximum RF exposure on the same point synchronously at the same time period is lower than for one single transmitter.”

A comparison of the variability of E‐field levels at different frequency bands as evaluated in this and in previously published studies (Manassas et al. 2012; Joseph et al. 2009; Joseph and Verloock 2010) was also attempted. The difficulties usually encountered in such comparisons (e.g., different measurement point selection criteria, considered time periods, measurement protocol and equipment, spectrum allocation, evaluation metrics) are also present here. In Table 3, the results of comparison with (Joseph et al. 2009) only are shown for brevity. The R ratio applied in Joseph et al. (2009) is also evaluated here to facilitate comparison. Although the differences mentioned above impose difficulties to a straightforward comparison, a good agreement is observed between the two studies.

Finally, trendlines of the mean E‐field levels per week over the considered time period, for the cellular frequency bands are plotted (Figure 7) and the statistical significance of individual sensors' trends is evaluated (Table 4). Trendlines are based on the mean value of the distribution of the weekly median E‐field values of the 13 monitoring sensors. An upward trend at the 3.6 GHz band, also shown in Figure 3, is present. This trend is considered significant since 12/13 sensors have a statistically significant increasing trend. At the same time an increasing trend at 1800 MHz is also observed. However, only 7 out of 13 sensors exhibit a statistically significant increasing trend (Table 4), which raises questions about the reliability of this observation. This is also the case for the other trends observed in Figure 7, either increasing (700, 800, 2100, 2600 MHz, CN's Total) or decreasing (900 MHz). The significant upward trend at 3.6 GHz band is also confirmed in ANFR's report (ANFR 2024). An upward trend of the total E‐field is also reported in ANFR (2024), also observed here, though not statistically significant. A limiting factor for the comparison of the two studies is the small number of measurement locations (i.e., 13) included in our study. Further studies are needed to provide a clearer view on the topic of environmental EMF levels during the 5G deployment phase.

The strengths of the current study, include an extensive data set of continuous (i.e., every 6‐min) frequency‐selective measurements, over a significant time period, spanning 112 weeks, that includes the rollout phase of 5G networks in Greece. The availability of such measurements at the 3.6 GHz band, where the newly introduced active antennas operate, offers an advantage in dealing with challenges in exposure assessment of such systems (e.g., time variability). However, the limited number of measurement locations and the selection criteria of these locations (i.e., rooftops of building at dense urban environments) limits comparability with similar studies, (ANFR 2024; Iakovidis et al. 2022) and makes it difficult to draw more general conclusions.

## 5 Conclusions

In this study, continuous measurements of E‐field from 13 monitoring sensors located in the five largest Greek cities and collected over the last 2 years were analyzed with a focus on the 3.6 GHz frequency band, where 5G networks operate. An increasing trend in environmental EMF levels at this band over these years was identified but the maximum registered incident power density remained well below the reference levels of ICNIRP and the Greek legislation. A considerably higher variation of exposure levels within a week at the 3.6 GHz band compared to other frequency bands was observed in terms of weekly maximum‐to‐median incident power ratio. This result highlights the necessity of extrapolation to the actual maximum when short‐time measurements (e.g., of 6 min) are used as a proxy for compliance assessment. However, this high maximum‐to‐median ratio considerably decreases by considering 30‐min averaging of the measurements, as suggested by the 2020 ICNIRP guidelines. Notably, the median E‐field values at the 3.6 GHz band are lower than those of legacy networks.

No significant trends at older frequency bands were identified possibly due to limited spatial sampling, with only 13 measurement locations. Therefore, further research with broader geographical coverage and additional monitoring sensors would be valuable to evaluate these trends of exposure in the context of 5G rollout.

## Conflicts of Interest

The authors declare no conflicts of interest.
